# Sequential 2.5 mg letrozole/FSH therapy is more effective for promoting pregnancy in infertile women with PCOS: a pragmatic randomized controlled trial

**DOI:** 10.3389/fendo.2023.1294339

**Published:** 2024-01-15

**Authors:** Li-Juan Chen, Yi Liu, Ling Zhang, Jing-Yi Li, Wen-Qian Xiong, Tao Li, Hui Ding, Bing-Jie Li

**Affiliations:** ^1^ Department of Obstetrics and Gynecology, Union Hospital, Tongji Medical College, Huazhong University of Science and Technology, Wuhan, Hubei, China; ^2^ Tongji Medical College, Huazhong University of Science and Technology, Wuhan, Hubei, China

**Keywords:** polycystic ovary syndrome, infertility, ovulation induction, letrozole, sequential therapy

## Abstract

**Study question:**

In infertile women with polycystic ovary syndrome (PCOS), is the sequential use of letrozole 2.5 mg/follicle stimulating hormone(FSH) more effective than letrozole 5 mg/FSH in stimulating ovulation and promoting pregnancy?

**Research design and methods:**

The study was designed as a prospective, single-center, randomized, controlled pragmatic clinical trial. 220 infertile women between the ages of 20 and 40, who matched the Rotterdam criteria for PCOS and had no other identified reasons for infertility were enrolled from April 2023 to July 2023.The participants were randomly assigned to two groups in a 1:1 ratio. One group received 2.5 mg of letrozole on cycle days 3-7 with a sequential injection of 75 IU FSH on cycle days 8-10 (n = 110), while the other group received 5 mg of letrozole on cycle days 3-7 with a sequential injection of 75 IU FSH on cycle days 8-10 (n = 110). The duration of FSH treatment varied depending on the follicular development stage. Each participant underwent one to three treatment cycles until achieving pregnancy.The primary outcome was the cumulative pregnancy rate of all the participants. Secondary outcomes included characteristics and clinical pregnancy rates of all the intervention cycles.

**Results:**

For all 220 participants, the sequential letrozole 2.5 mg/FSH treatment group had a significantly higher cumulative pregnancy rate compared to the letrozole 5 mg/FSH treatment group (72.7% versus 59.1%, RR (95%CI) = 1.23 (1.02, 1.49), P-value = 0.033). For all 468 intervention cycles, letrozole 2.5 mg/FSH group had a significantly higher clinical pregnancy rate than the letrozole 5 mg/FSH group (36.2% versus 26.3%, P-value = 0.021), no statistically significant differences were observed in ovulation rates or adverse effects.

**Conclusions:**

The data indicate that the sequential letrozole 2.5mg/FSH protocol may be more effective than the sequential letrozole 5mg/FSH protocol for promoting pregnancy in infertile women with PCOS.

**Clinical trial registration:**

www.chictr.org.cn, identifier ChiCTR2300069638.

## 1 Introduction

Polycystic ovary syndrome (PCOS) is an endocrine condition that is commonly characterized by persistent anovulation, hyperandrogenism, and insulin resistance in women of gestational age (1, 2). PCOS is the most prevalent cause of anovulatory infertility in women, accounting for approximately 75% of anovulatory infertility, with a prevalence rate between 5% and 10% (3). Ovulation abnormalities are responsible for roughly one quarter of all cases of infertility in couples (4). PCOS is the most prevalent reason for anovulatory infertility, accounting for about 70 percent of the overall cases (5). For PCOS patients, the standard method of treatment for anovulatory infertility is restoring mono-ovulation (6). Consequently, ovulation induction is the most crucial treatment option for infertile patients with PCOS, despite various therapeutic medications have varying ovulation induction effects (7).

There are currently primarily three types of medications for PCOS patients to induce ovulation (8–10): (i) Clomiphene citrate (CC), which has been used as a first-line ovulation induction drug for PCOS patients for decades, approximately 20% to 25% of patients with PCOS still have CC resistance in clinical practice. Besides, it has some drawbacks, including a long half-life and the occupation of estrogen receptors, which can have negative effects on the endometrium and cervical mucus and result in unsatisfactory pregnancy rates. Additionally, Clomiphene citrate is associated with a 10% increased risk of multiple pregnancies, while hyperstimulation syndrome is uncommon, which makes its clinical application subject to some restrictions (11). (ii) Letrozole (LE), which as a third-generation steroidal aromatase inhibitor, reversibly binds to aromatase enzyme and inhibits estrogen production (12). By reducing the process of converting androgens to estrogens, it lowers the body’s estrogen levels, relieves the negative feedback inhibition on the hypothalamus-pituitary gland, and increases the secretion of gonadotropin-releasing hormone (GnRH), which contributes to the development of follicles, and has the advantages of a shorter half-life and no effect on estrogen receptors compared with CC. It has gotten a lot of interest since Mohamed et al. used LE for the first time to promote ovulation and achieved good clinical efficacy (13). (iii) Follicle stimulating hormone (FSH), which activates aromatization of androgens to estrogen by granulosa cells and follicle maturation, acts on the ovary to help grow and mature small follicles (14). Although FSH can be used to treat CC-resistant PCOS patients, its tendency to induce the development of multiple follicles at the same time, which increases the incidence of multiple pregnancies and the incidence of ovarian hyperstimulation syndrome (OHSS), and its price is relatively expensive, which limits its use in clinical practice (15).

Since the highest pregnancy rate with the fewest problems is considered to be the optimal outcome of ovulation induction, the procedure was accomplished by administering consecutive injections of FSH after receiving letrozole or other medications (16). This procedure has been referred to as minimal stimulation, and Kistner RW initially proposed the concept of minimal stimulation (17). It has been demonstrated to raise overall pregnancy rates while reducing adverse consequences; notably, ovarian hyperstimulation syndrome rarely occurs (18).

Studies have continued over the last few decades employing a variety of drugs together with varying gonadotrophin dosages and types (19). Despite the diversity of prior research, which ranged from retrospective evaluations to prospective randomized controlled trials, combination procedures have yielded clinical pregnancy rates equivalent to or greater than gonadotrophin-only therapies with less gonadotrophin usage and fewer multi-ovulation (20–23).

Even though numerous studies on the optimal dose of letrozole have been conducted in recent years, the optimal dose of letrozole for inducing ovulation remains debatable (24). Moreover, the most common doses of letrozole are 2.5 mg, 5 mg, and 7.5 mg, with a few reports of doses as high as 20 mg (25, 26). We analyzed the data of previously reported clinical trials on letrozole for inducing ovulation. Some studies concluded that 5 mg was more effective than 2.5 mg inducing ovulation, whereas others determined that 2.5 mg was more efficient than 5 mg and 7.5 mg in promoting ovulation (27–29).

Our recent clinical trial using sequential letrozole/gonadotrophin to induce ovulation in infertile women with PCOS identified that the sequential protocol’s ovulation rate and live birth rate are higher than the letrozole protocol (20). In the present pragmatic clinical trial study, we aim to identify the optimal dose of letrozole by comparing the ovulation rate, pregnancy rate, and adverse effects of two doses of letrozole in sequential letrozole/FSH therapy.

## 2 Materials and methods

### 2.1 Trial design

This pragmatic clinical trial was performed at Union Hospital of Tongji Medical College, Huazhong University of Science and Technology, to compare treatment outcomes of different letrozole doses for ovulation induction in infertile women with PCOS, using sequential letrozole/FSH. Each individual paid for their own medication and examination, and no extra compensation was offered. The study was approved by the Medical Ethics Committee of Tongji Medical College, Huazhong University of Science and Technology (approval number 0002-02; approval date March 5, 2023), and it was registered on the Chinese Clinical Trial Registration website (www.chictr.org.cn; identifier ChiCTR2300069638; registration date March 22, 2023). Before participation, signed informed consent with self-signature was obtained from each participant. Full details of the trial protocol can be found in the Chinese Clinical Trial Registry, available at https://www.chictr.org.cn/showprojEN.html?proj=193178.

### 2.2 Participants

The first participant became involved on 3 April 2023, and the final participant accomplished the study on 31 July 2023. Outpatients with PCOS and infertility who desired to conceive were evaluated using inclusion and exclusion criteria to identify those who were suitable and had no other definite causes of infertility. Inclusion criteria involved: (i) PCOS patients who met the Rotterdam criteria in 2003 (diagnosis can be created if two of three are present): oligo-ovulation or anovulation, clinical or biochemical hyperandrogenemia, polycystic ovary/ies changes under ultrasound (Rotterdam ESHRE/ASRM Sponsored PCOS Consensus Workshop Group, 2004) (30); (ii) 20 - 40 years old, normal intercourse without protection, duration of infertility longer than 1 year; (iii) hysterosalpingography indicates that normal uterine morphology and at least one Fallopian tube is unobstructed; (iv) the sperm analysis of the spouse shows no abnormality; (v) normal organ function without any endocrine disease such as hypopituitarism, hyperthyroidism, hyperprolactinemia, congenital adrenal hyperplasia, Cushing’s syndrome, tumor of ovaries or adrenal gland, etc. Exclusion criteria involved: allergy to relevant drugs, failure to cooperate, severe cardiovascular disease, severe defects of liver or kidney function, pregnancy or lactation, previous history of abnormal uterine or uterine cavity disease, and previous contact with mutagenic toxins and radiation.

### 2.3 Intervention

Outpatients who met the inclusion and exclusion criteria for this research were selected as potential candidates for this study. Prior to their participation, the researcher provided a description of the study’s purpose, methodology, potential benefits, and risks. Participants were, only after they signed the informed consent form, recruited for the study. A computer-generated random number table was used to allocate eligible participants into two groups (Group A and Group B) in a 1:1 ratio. Group A participants (letrozole 2.5 mg/FSH group) received a daily dosage of 2.5 mg letrozole (Furui, Jiangsu Hengrui Medicine Co., Ltd., Lianyungang, China) from cycle days 3 to 7, followed by a sequential therapy of 75 IU FSH (urofollitropin, Lizhu Pharmaceutical Factory, Zhuhai, China) daily from cycle days 8 to 10. Participants in Group B (letrozole 5 mg/FSH group) were given 5 mg of letrozole daily from cycle days 3 to 7, followed by sequential therapy with 75 IU of FSH daily on cycle days 8 to 10. The duration of FSH administration varies according to follicular development. Vaginal B-ultrasound was utilized to monitor follicular development beginning on day 11 of the cycle. During scanning, the number/size of follicles and endometrial thickness were measured. Blood was drawn to detect the sex hormone concentration when at least one dominant follicle reached ≥ 18 mm, and then 10,000 IU human chorionic gonadotrophin (HCG) was injected intramuscularly to trigger ovulation. Participants were advised to engage in intercourse 24 to 36 hours later. In order to monitor ovulation, a vaginal B-ultrasound was conducted 48 hours after HCG injection. Two weeks of Dydrogesterone were consumed orally. Quantitative HCG testing was carried out two weeks later to diagnose conception, and B-ultrasound was conducted four weeks later to diagnose clinical pregnancy. Each participant underwent one to three cycles of treatment until pregnancy was achieved.

### 2.4 Outcome measures

The primary outcome was the cumulative pregnancy rate for all participants. Secondary outcomes included the characteristics and clinical pregnancy rates of all intervention cycles. These characteristics were listed as follows: (i) ovulation characterized by a decrease in ovarian follicles and fluid in the pouch of Douglas, detected through B-mode ultrasonography 2 days after HCG administration; (ii) cancellation cycle referred to a cycle that has been discontinued due to premature ovulation, follicular dysplasia, or ovarian hyperstimulation syndrome (OHSS); (iii) biochemical pregnancy defined as a positive β-HCG pregnancy test 2 weeks after ovulation, clinical pregnancy defined as the observation of a gestational sac with fetal echoes and primitive cardiac tube pulsation inside the uterine cavity via B-ultrasound 2 weeks after a positive pregnancy test, singleton pregnancy (the sample for singleton pregnancies was derived from ongoing pregnancies), multiple pregnancy, early abortion(gestational age ≤ 14 weeks), ectopic pregnancy; (iv) duration of FSH treatment; (v) rate of one mature follicle (diameter ≥ 18 mm) and ovulation with one follicle; (vi) days required for follicles to mature and ovulate; (vii) sex hormone concentration on HCG injection day; (viii) endometrial thickness on HCG injection day and ovulation day.

### 2.5 Sample size

The number of samples estimated by G*Power 3.1 software was 91 subjects per group with a two-sided probability value of 0.05 and 0.9 statistical power using Pearson’s chi-squared test, based on the findings of the previous studies (31, 32). In consideration of non-compliance and loss to follow-up, the ultimate sample size per group was 110 after correction, allowing for a dropout rate of 17%.

### 2.6 Randomization and masking

Using SPSS software (Version 26, IBM, Armonk, NY), a statistician generated a randomization scheme that allocated the enrolled participants in a 1:1 ratio to two groups. The randomization scheme consisted of an enrollment order combined with treatment codes (A and B). Participants were assigned by a clinician who was aware of the order of the randomization scheme but unaware of the meaning behind each code. The assignment was made based on the enrollment order specified in the randomization scheme. Sonographers, statisticians, and outcome evaluators remained blinded to the allocation, while physicians and patients were informed.

### 2.7 Statistical analysis

The intervention cycle (IC) analysis comprised all intervention cycles, while the complete data (CD) analysis comprised only those intervention cycles for which complete data were collected. Statistical analysis was conducted using SPSS for Windows (Version 26). For qualitative (categorical) data, the outcome was expressed as number of cases and percentage, and differences between the two groups were analyzed using chi-squared test at a two-sided significance value of 0.05. Fisher’s exact test was used if the total number of cases was less than 40, or if a theoretical frequency was equal to 0, or if two theoretical frequencies were greater than or equal to 1 but less than 5.If the total number of cases was greater than 40, a theoretical frequency was greater than or equal to 1 but less than 5, P-value required continuity correction. For quantitative (numerical) variables, the Shapiro-Wilke test for a single sample was used to evaluate the normality of distribution. In the case of normally distributed data, Student’s t-test was performed to two independent samples, and the mean ± standard deviation was presented. The Mann-Whitney U-test was utilized to asymmetrical data presented as a median and interquartile range. A stratification analysis was performed when evaluating cumulative pregnancy rate and clinical pregnancy rate. A degree of significance of less than or equal to 0.05 was considered statistically significant. Figures were generated using GraphPad Prism 9 software.

## 3 Results

### 3.1 Participation flow chart

**Figure 1:**
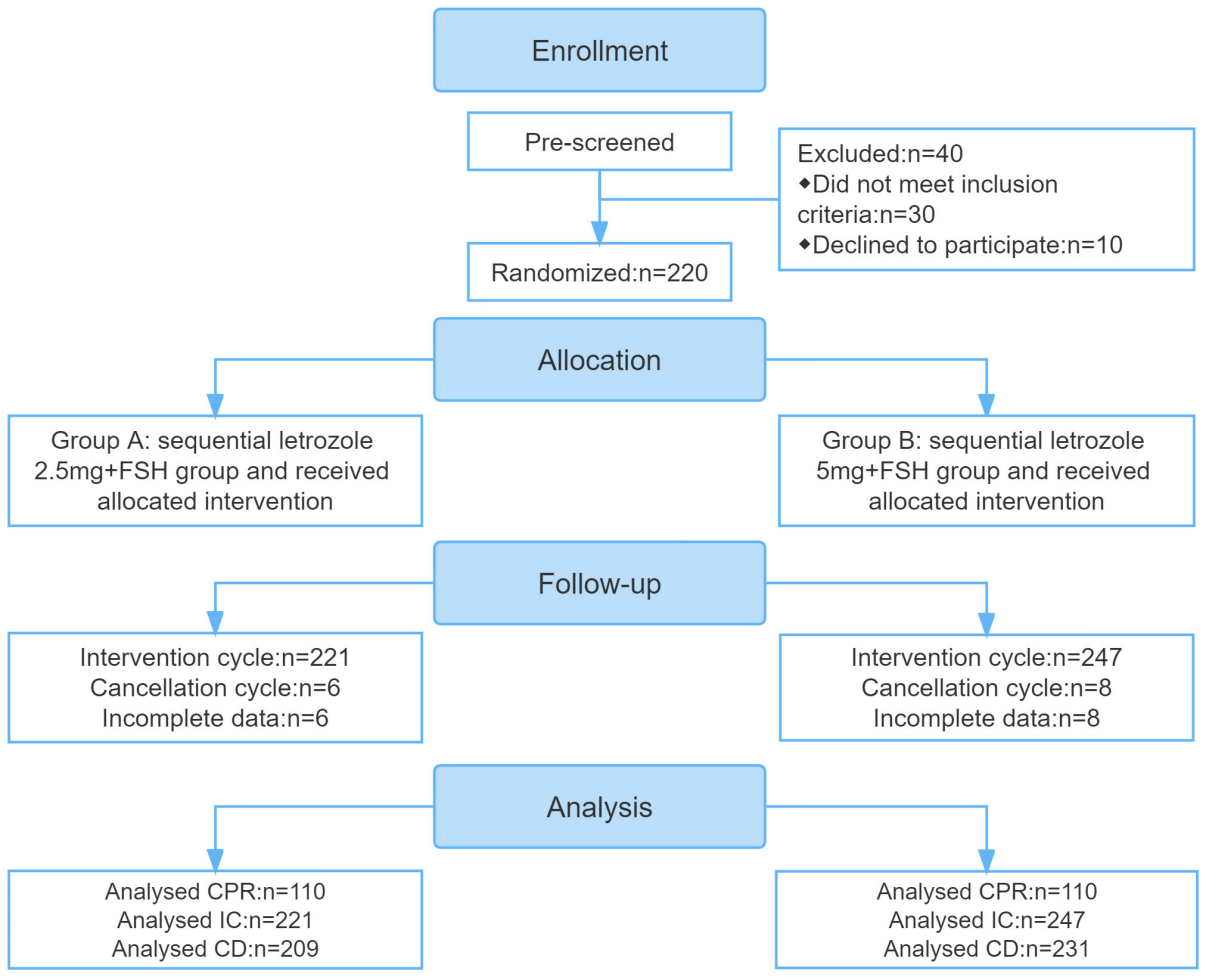
CONSORT flow diagram revealing the participation process for each stage of the study. CONSORT (Consolidated Standards of Reporting Trials); FSH, follicle stimulating hormone; CPR, cumulative pregnancy rate; IC, intervention cycle; CD, complete data.

The complete procedure for study participants is depicted in **Figure 1**. Two hundred twenty participants were randomly assigned from 230 invited women who were initially considered suitable; 110 were assigned to the letrozole 2.5 mg/FSH group, and 110 were assigned to the letrozole 5 mg/FSH group. All 220 participants completed the process from corresponding treatment to pregnancy evaluation; they became included in the CPR analysis. Fourteen out of 468 intervention cycles were canceled, 14 cycles had incomplete data recordings, thus 440 cycles were included in the complete data analysis.

### 3.2 Baseline data

**Table 1** depict baseline demographic, clinical and endocrine characteristics. No significant differences existed between the two groups.

**Table 1:** Baseline characteristics of participants.

Characteristic	Group A (n = 110) Letrozole 2.5 mg + FSH	Group B (n = 110) Letrozole 5 mg + FSH	*P*-value
Age, years	28(26~30)	28(26~29)	0.554
BMI, kg/m^2^	23.24(21.56~25.69)	23.4(21.45~25.7)	0.96
HOMA-IR	2.65(1.86~3.62)	2.66(1.89~3.59)	0.654
Sex hormone			
LH, IU/L	8.56(5.89~10.66)	8.13(4.75~11.64)	0.523
FSH, IU/L	6.78(5.91~7.74)	6.53(5.58~7.51)	0.362
LH/FSH	1.2(0.93~1.57)	1.24(0.72~1.8)	0.841
PRL, ng/ml	18.65(13.49~24.15)	16.11(12.2~22.37)	0.132
AMH, ng/ml	8.25(5.9~10.07)	8.4(5.85~12.29)	0.158
Stratification			
Age≤ 28, years	64(58.2%)	69(62.7%)	0.491
BMI<24, kg/m^2^	70(63.6%)	64(58.2%)	0.407
No insulin resistance	82(74.5%)	83(75.5%)	0.876
Primary infertility	94(85.5%)	91(82.7%)	0.58
LH ≤ 12, IU/L	93(84.5%)	84(76.4%)	0.126
FSH ≤ 8, IU/L	88(80%)	92(83.6%)	0.484
LH/FSH<2	97(88.2%)	92(83.6%)	0.333
PRL ≤ 30, ng/ml	96(87.3%)	97(88.2%)	0.837
T ≤ 2, nmol/L	104(94.5%)	101(91.8%)	0.422
AMH>4, ng/ml	100(90.9%)	104(94.5%)	0.299

Values are presented as n (%) or median (interquartile range). P-values were evaluated using chi-squared test for qualitative data and Mann-Whitney U-test for quantitative data. FSH, follicle stimulating hormone; BMI, body mass index; HOMA-IR, homeostasis model assessment of insulin resistance; LH, luteinizing hormone; PRL, prolactin; AMH, anti-Müllerian hormone; T, testosterone.

### 3.3 Primary outcome

**Table 2** depicts cumulative pregnancy rate among participants. The cumulative pregnancy rate was substantially higher in Group A than in Group B (72.7% versus 59.1%, RR (95%CI) = 1.23 (1.02, 1.49), P-value = 0.033). Age>28 years, BMI ≥24 kg/m^2^, LH ≤ 12IU/L, FSH ≤ 8IU/L, PRL ≤ 30ng/ml, T ≤ 2nmol/L and AMH>4ng/ml were subgroups where the positive effect of Group A on cumulative pregnancy rate was evident (P-value<0.05).

**Table 2:** Cumulative pregnancy rate of participants.

Item		Group A, n(%) Letrozole 2.5 mg + FSH	Group B, n(%) Letrozole 5 mg + FSH	RR (95%CI)	*P*-value
CPR		80/110(72.7%)	65/110(59.1%)	1.23(1.02,1.49)	0.033
Age, years	≤ 28	45/64(70.3%)	42/69(60.9%)	1.16(0.9,1.48)	0.253
	> 28	35/46(76.1%)	23/41(56.1%)	1.36(0.99,1.86)	0.048
BMI, kg/m^2^	< 24	53/70(75.7%)	44/64(68.8%)	1.1(0.89,1.36)	0.368
	≥ 24	27/40(67.5%)	21/46(45.7%)	1.48(1.01,2.17)	0.042
Insulin resistance	No	61/82(74.4%)	52/83(62.7%)	1.19(0.96,1.46)	0.105
	Yes	19/28(67.9%)	13/27(48.1%)	1.41(0.88,2.25)	0.139
Infertility	Primary	69/94(73.4%)	56/91(61.5%)	1.19(0.97,1.46)	0.085
	Secondary	11/16(68.8%)	9/19(47.4%)	1.45(0.81,2.59)	0.306^a^
LH, IU/L	≤ 12	68/93(73.1%)	49/84(58.3%)	1.25(1.01,1.56)	0.038
	> 12	12/17(70.6%)	16/26(61.5%)	1.15(0.75,1.77)	0.543
FSH, IU/L	≤ 8	66/88(75%)	54/92(58.7%)	1.28(1.04,1.58)	0.02
	> 8	14/22(63.6%)	11/18(61.1%)	1.04(0.64,1.69)	0.87
LH/FSH	< 2	72/97(74.2%)	56/92(60.9%)	1.22(1,1.49)	0.05
	≥ 2	8/13(61.5%)	9/18(50%)	1.23(0.66,2.31)	0.711^a^
PRL, ng/ml	≤ 30	70/96(72.9%)	56/97(57.7%)	1.26(1.02,1.56)	0.027
	> 30	10/14(71.4%)	9/13(69.2%)	1.03(0.63,1.69)	> 0.1^a^
T, nmol/L	≤ 2	76/104(73.1%)	59/101(58.4%)	1.25(1.02,1.53)	0.027
	> 2	4/6(66.7%)	6/9(66.7%)	1(0.48,2.08)	> 0.1^a^
AMH, ng/ml	≤ 4	6/10(60%)	3/6(50%)	1.2(0.47,3.09)	> 0.1^a^
	> 4	74/100(74%)	62/104(59.6%)	1.24(1.02,1.51)	0.029

a P-values were calculated using Fisher’s exact test. RR, relative risk; CI, confidence interval; CPR, cumulative pregnancy rate.

### 3.4 Secondary outcomes

Fourteen out of 468 intervention cycles were canceled, while 14 cycles had incomplete data recordings; thus, a total of 440 cycles were included in the complete data analysis. **Table 3** illustrate the characteristics of these cycles. For all the intervention cycles, there were no significant differences observed between the two groups in terms of ovulation rate, cancellation rate, incidence of early ovulation, follicular dysplasia, and OHSS. However, Group A exhibited significantly higher rates of biochemical (40.7% versus 28.3%, P-value = 0.005) and clinical pregnancy (36.2% versus 26.3%, P-value = 0.021).

**Table 3:** Cycle characteristics.

Characteristic	Group A Letrozole 2.5 mg + FSH	Group B Letrozole 5 mg + FSH	*P*-value
**Intervention cycle analysis**	n=221	n=247	
Ovulation rate	217/221(98.2%)	239/247(96.8%)	0.329
Cancellation rate	6/221(2.7%)	8/247(3.2%)	0.740
Ovulate prematurely	2/221(0.9%)	0/247(0%)	0.222^a^
Follicular dysplasia	2/221(0.9%)	3/247(1.2%)	>0.1^a^
OHSS	0/221(0%)	1/247(0.4%)	>0.1^a^
Reproductive outcomes overall			
Biochemical pregnancy	90/221(40.7%)	70/247(28.3%)	0.005
Clinical pregnancy	80/221(36.2%)	65/247(26.3%)	0.021
Singleton pregnancy	60/80(75%)	46/65(70.8%)	0.568
Multiple pregnancy	10/80(12.5%)	14/65(21.5%)	0.145
Early abortion	9/80(11.3%)	4/65(6.2%)	0.285
Ectopic pregnancy	1/80(1.3%)	1/65(1.5%)	>0.1^a^
**Complete data analysis**	n=209	n=231	
Days of uFSH treatment	4.47 ± 0.15	4.51 ± 0.14	0.852
One mature follicle	169/209(80.9%)	149/231(64.5%)	<0.01
Ovulation with one follicle	152/209(72.7%)	154/231(66.7%)	0.168
Days required for follicles to mature	13.3 ± 0.16	13.12 ± 0.14	0.373
Days required for follicles to ovulation	15.51 ± 0.16	15.27 ± 0.15	0.202
Hormone concentration on HCG injection day			
LH, IU/L	10.14(7.54~14.35)	8.86(6.35~13.1)	0.014
Oestradiol, pg/ml	228(156.35~355)	188.5(120.55~302.5)	0.001
Progesterone, ng/ml	0.23(0.1~0.38)	0.26(0.16~0.49)	0.011
Endometrial thickness, mm			
HCG injection day	9.16 ± 0.14	9.36 ± 0.15	0.367
Ovulation day	10.16 ± 0.14	10.67 ± 0.15	0.015

a P-values were calculated using Fisher’s exact test. Values are showed as n (%) or median (interquartile range). When the medians are equal, values are showed as mean ± SD.

Moreover, Group A showed a significantly higher rate of obtaining only one mature follicle compared to Group B (80.9% versus 64.5%, P-value <0.01), whereas there was no significant difference in the ovulation rate with one follicle (72.7% versus 62.7%, P-value >0.05). The duration of FSH treatment (4.47 ± 0.15 versus 4.51 ± 0.14, P-value >0.05) and days required for follicles to mature (13.3 ± 0.16 versus 13.12 ± 0.14, P-value >0.05) or ovulate (15.51 ± 0.16 versus 15.27 ± 0.15, P-value >0.05) were similar between both groups. The hormone concentration on HCG injection day significantly differed between the two groups. The endometrial thickness on ovulation day was significantly less in Group A (P-value<0.05), whereas there was no substantial difference on the day of HCG administration.

**Table 4** displays the clinical pregnancy rate for the intervention cycle with complete data recorded. The clinical pregnancy rate in Group A was significantly greater than in Group B (38.3% versus 27.7%, RR (95%CI) = 1.38(1.05,1.81), P-value = 0.018). The beneficial effect of Group A on clinical pregnancy rate was apparent (P-value<0.05) in subgroups with ages >28 years old, cycles with only one mature follicle and endometrial thickness on ovulation day>10 mm. There was no significant difference between groups for participants who received treatment once; however, for participants who received treatment for 2 or more cycles, Group A had a significantly higher clinical pregnancy rate (P-value<0.05).

**Table 4:** Clinical pregnancy rate of complete data cycles.

Item		Group A, n(%) Letrozole 2.5 mg + FSH	Group B, n(%) Letrozole 5 mg + FSH	RR (95%CI)	*P*-value
**Complete data analysis**		n=209	n=231		
Clinical pregnancy rate		80/209(38.3%)	64/231(27.7%)	1.38(1.05,1.81)	0.018
Age,years	≤ 28	45/119(37.8%)	41/139(29.5%)	1.28(0.91,1.81)	0.158
	> 28	35/90(38.9%)	23/92(25%)	1.56(1,2.41)	0.044
BMI, kg/m^2^	< 24	53/137(38.7%)	44/134(32.8%)	1.18(0.86,1.62)	0.315
	≥ 24	27/72(37.5%)	20/97(20.6%)	1.82(1.11,2.97)	0.015
One mature follicle	No	15/40(37.5%)	23/82(28%)	1.34(0.79,2.27)	0.29
	Yes	65/169(38.5%)	41/149(27.5%)	1.4(1.01,1.93)	0.039
Hormone concentration on HCG injection day					
LH, IU/L	≤ 10	40/104(38.5%)	37/136(27.2%)	1.41(0.98,2.04)	0.064
	> 10	40/105(38.1%)	27/95(28.4%)	1.34(0.9,2)	0.148
Oestradiol, pg/ml	≤ 200	25/82(30.5%)	24/122(19.7%)	1.55(0.95,2.52)	0.076
	> 200	55/127(43.3%)	40/109(36.7%)	1.18(0.86,1.62)	0.302
Progesterone, ng/ml	≤ 0.25	47/119(39.5%)	34/113(30.1%)	1.31(0.92,1.88)	0.133
	> 0.25	33/90(36.7%)	30/118(25.4%)	1.44(0.96,2.18)	0.08
Endometrial thickness on ovulation day, mm	≤ 10	40/130(30.8%)	30/119(25.2%)	1.22(0.82,1.83)	0.33
	> 10	40/79(50.6%)	34/112(30.4%)	1.67(1.17,2.38)	0.005
Cycle	1	40/106(37.7%)	31/105(29.5%)	1.28(0.87,1.88)	0.207
	2~3	40/103(38.8%)	33/126(26.2%)	1.48(1.01,2.17)	0.041

## 4 Discussion

Our present study in infertile women with PCOS revealed that letrozole 2.5 mg/FSH sequential therapy was superior at ovulation induction and pregnancy rates than letrozole 5 mg/FSH sequential therapy. Specifically, there were no notable disparities in adverse drug reactions.

Currently, the majority of frequently used to stimulate ovulation are clomiphene, letrozole, and FSH, with FSH being the most effective and clinically valuable (33). Nevertheless, FSH is associated with an increased risk of multiple pregnancies and OHSS (34). Letrozole is an aromatase inhibitor of the third generation, and its effects on the human body are primarily reflected in two aspects (35). Firstly, aromatase inhibition diminishes estrogen production, which relieves estrogen’s inhibiting effect on the hypothalamus and pituitary gland, resulting in the release of FSH and LH and an increase in follicular recruitment (36). Secondly, the increase of androgens in the ovary elevates the sensitivity of insulin-like growth factor, which together with FSH, promotes follicular development (37).

Letrozole is associated with comparable ovulation rates and reduced rates of complications such as multiple births and OHSS than clomiphene. The 2018 European Society of Human Reproduction and Embryology (ESHRE) “International Evidence-Based Guidelines for the Evaluation and Management of Polycystic Ovarian Syndrome” recommends letrozole as a first-line ovulation induction agent for infertile patients with PCOS (38). Letrozole is the initial treatment option for infertile patients with PCOS (39). Obstetricians and gynecologists tend to use urinary follicle-stimulating hormone (uFSH) in combination with letrozole for ovulation induction in infertile women in order to reduce the risk of the aforementioned adverse events and reduce the financial burden (40).

Our recent report suggested that sequential letrozole/gonadotrophin is superior to letrozole alone for inducing ovulation and facilitating pregnancy in infertile women with PCOS (20). Sequential letrozole/gonadotrophin is recommended as a high-reward, low-risk ovulation promotion regimen. There is disagreement regarding the optimal dose of letrozole in the clinical treatment of PCOS. Previous investigations have demonstrated that 5 mg/day of letrozole is more efficacious than 2.5 mg/day but that 7.5 mg/day has no advantage over 5 mg/day (41, 42). Other studies have suggested that the efficacy of the 2.5 mg dose is more confident and promotes ovulation more effectively than the 5 mg dose and the higher 7.5 mg dosage (31, 43). The present study found that letrozole at 2.5 mg/day was more effective than letrozole at 5 mg/day. The current investigation revealed that the utilization of sequential therapy involving letrozole 2.5 mg/FSH did not necessitate an escalation in the dosage of FSH in order to attain an equivalent therapeutic outcome. Moreover, the synergistic effect of FSH made it possible to achieve a better therapeutic effect with 2.5 mg of letrozole without the need to increase the letrozole dosage additionally.

In this study, there was no statistically significant difference in fundamental characteristics between the letrozole 2.5mg/FSH group and the letrozole 5mg/FSH group. However, the incidence of single follicular development was statistically significantly higher in the former group, with a 16.4% absolute difference. Statistically significant improvements were also observed in the biochemical and clinical pregnancy rates in the letrozole 2.5 mg/FSH group. Furthermore, this analysis was stratified by age and body mass index (BMI) (44). The group receiving letrozole 2.5 mg/FSH had a statistically significant higher cumulative pregnancy rate among patients older than 28. The cumulative pregnancy rate in the letrozole 5 mg/FSH group was considerably lower in patients over 28 years of age compared to patients under 28. This indicates letrozole 2.5 mg/FSH is more suitable for patients over 28.

A large BMI is disadvantageous to ovulation in infertile patients with PCOS, and treatment with ovulation-promoting medications is less effective in obese patients than in patients with a normal BMI (45, 46). In the current study, the cumulative pregnancy rate decreased in both groups of patients with BMI of 24; however, the decrease was more pronounced in the BMI-stratified letrozole 5 mg/FSH group. The cumulative pregnancy rate was statistically higher in the letrozole 2.5 mg/FSH group for patients with a BMI below 24. Therefore, the letrozole 2.5 mg/FSH protocol is suggested for ovulation in patients with a BMI less than 24. It has also demonstrated that the letrozole 2.5 mg/FSH regimen is superior concerning pregnancy rate.

We investigate further why the letrozole 2.5 mg/FSH group in this study performed better regarding pregnancy rate. The single mature follicle development rate was significantly higher in the letrozole 2.5 mg/FSH group, suggesting that multiple follicle development is more likely to occur in the letrozole 5 mg/FSH group. It may be because a higher dose of letrozole inhibits the conversion of androgens to estrogens in the body to a greater extent (47, 48). This decrease in estrogen contributes to the discharge of the negative feedback inhibition of the hypothalamic-pituitary gland, increasing gonadotropin-releasing hormone (GnRH) secretion and, ultimately, the development of multiple follicles (49, 50). Group B, which had a high incidence of considerable follicle development, had statistically lower estrogen levels than group A on the day of HCG injection. We hypothesized that this could be due to the elevated rate of multiple follicle development in group B. We further speculate that the inferior egg quality in group B, compared to group A, ultimately leads to a low pregnancy rate and poor embryo quality. This possible explanation is consistent with the earlier observations that specifically examined egg quality and pregnancy outcome (51, 52).

The benefit of the present research was that, with the exception of the ovulation therapy, there was no disparity between the two groups regarding the other treatments, ranging from pretreatment to dydrogesterone application or the baseline characteristics. Consequently, the two treatment groups are identical at this point. The diagnostic criteria depend on the standard PCOS diagnostic criteria (53), which can be employed as a guide for treating the same group of patients in various clinical environments.

The present study has limitations that should be acknowledged. This study was open-label, so neither participants nor physicians were uninformed of the medication regimen. However, the patient assignment was determined by a computer-generated randomization algorithm implemented by a statistician who had no responsibility for patient enrolment (54). All selected outcomes were objective indicators, with the exception of B-ultrasound, which was administered by a physician who was oblivious to the treatment. There was no selection bias because the investigators did not know which group the next subject would be assigned to (55, 56). The preceding should reduce any bias induced by the open-label design of the study. When patients were aware of their own regimen and drug side effects, a bias toward side effects may have been introduced.

Nonetheless, this bias should have a tendency to cause more adverse events in sequential groups, which is contrary to the majority of adverse events observed here. Considering the existence of bias, the actual adverse events of the sequential group should be lower than the current information, which is more advantageous to the present conclusion (57, 58). Consequently, it could be stated that the subjective perception bias for adverse effects had little impact on the conclusion of the present investigation.

## 5 Conclusion

In conclusion, the current clinical trial demonstrated that letrozole 2.5mg/FSH was preferable to letrozole 5 mg/FSH for inducing ovulation and promoting pregnancy in infertile women with PCOS. Therefore, letrozole 2.5 mg/FSH is recommended as a superior protocol for ovulation induction. Consequently, in clinical practice, the starting dose of letrozole for ovulation induction is suggested to be 2.5 mg. If this dosage proves ineffective, alternative therapeutic regimens should be considered rather than increasing the dose of letrozole since it does not enhance the patient’s cycle pregnancy rate.

## Data availability statement

The original contributions presented in the study are included in the article/supplementary material. Further inquiries can be directed to the corresponding author.

## Ethics statement

The studies involving humans were approved by The Medical Ethics Committee of Tongji Medical College of Huazhong University of Science and Technology. The studies were conducted in accordance with the local legislation and institutional requirements. The participants provided their written informed consent to participate in this study.

## Author contributions

L-JC: Conceptualization, Data curation, Methodology, Project administration, Supervision, Writing – original draft, Writing – review & editing. YL: Conceptualization, Data curation, Methodology, Project administration, Supervision, Writing – review & editing. LZ: Data curation, Writing – review & editing. J-YL: Data curation, Investigation, Writing – review & editing. W-QX: Data curation, Investigation, Writing – review & editing. TL: Data curation, Writing – review & editing. HD: Data curation, Writing – review & editing. B-JL: Data curation, Formal analysis, Project administration, Validation, Visualization, Writing – original draft, Writing – review & editing.
